# Supplementary material for: Flavin Adenine Dinucleotide Fluorescence as an Early Marker of Mitochondrial Impairment During Brain Hypoxia
Source: Int J Mol Sci. 2020 Jun 1;21(11):3977. doi: 10.3390/ijms21113977 (PMC7312830; doi:10.3390/ijms21113977)
Supplement: Supplementary file 1 [file ijms-21-03977-s001.pdf]

Supplementary Material

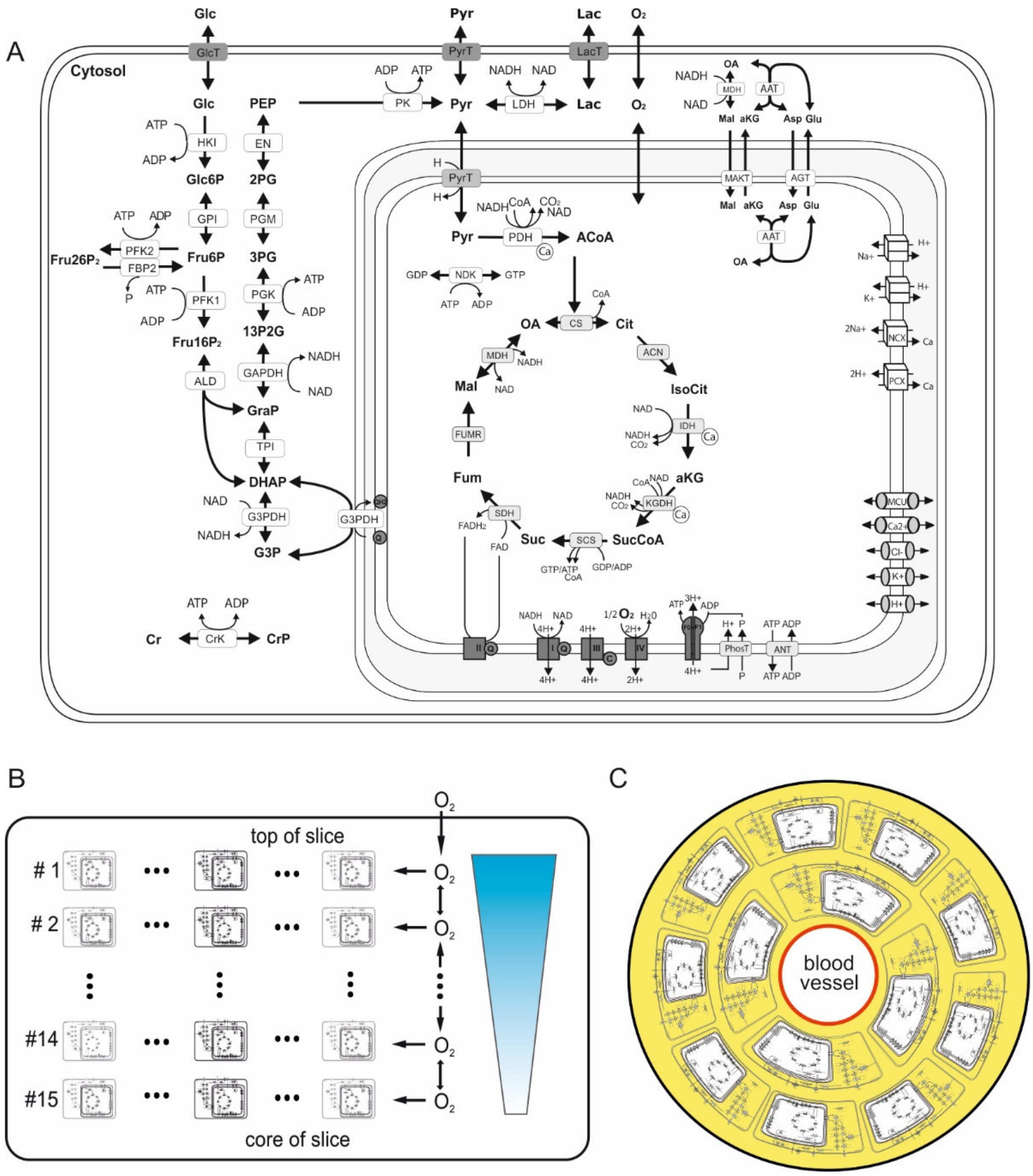

Supplementary Figure 1. A: Schematic representation of the kinetic model of neuronal energy metabolism: The model comprises the glycolytic pathway describing the conversion of glucose to pyruvate/lactate including the formation of the regulator fructose 2,6-bisphosphate (Fru26p); the malate-aspartate shuttle; the glycerol-3-phosphate shuttle; the citric acid cycle; the respiratory chain; the mitochondrial electrophysiology including sodium, potassium, chloride, protons and calcium; the mitochondrial ATP generation. B: Schematic representation of the tissue model in the in vitro condition: The tissue model dissects the slice from the top to the slice core into a series of 15 thin layers. Assuming spatial isotropy in cell layers parallel to slice surface, all neuronal cells resident in a given layer are represented by a single cell so that the three-dimensional slice can approximated by a one-dimensional row of cells, which are diffusively coupled. The metabolism of the cell representing one layer of the slice is described by the kinetic model of neuronal energy metabolism. Oxygen diffuses from the top to the slice core. C: Schematic representation of the tissue model for the in vivo condition: The tissue model dissects the tissue in 10 concentric hollow cylinders around a central blood vessel. Oxygen supply is modeled by diffusion between these cylinders and the blood vessel. Each cylinder is equipped with the single-cell metabolic system. The figure is reproduced with few modifications from [39]

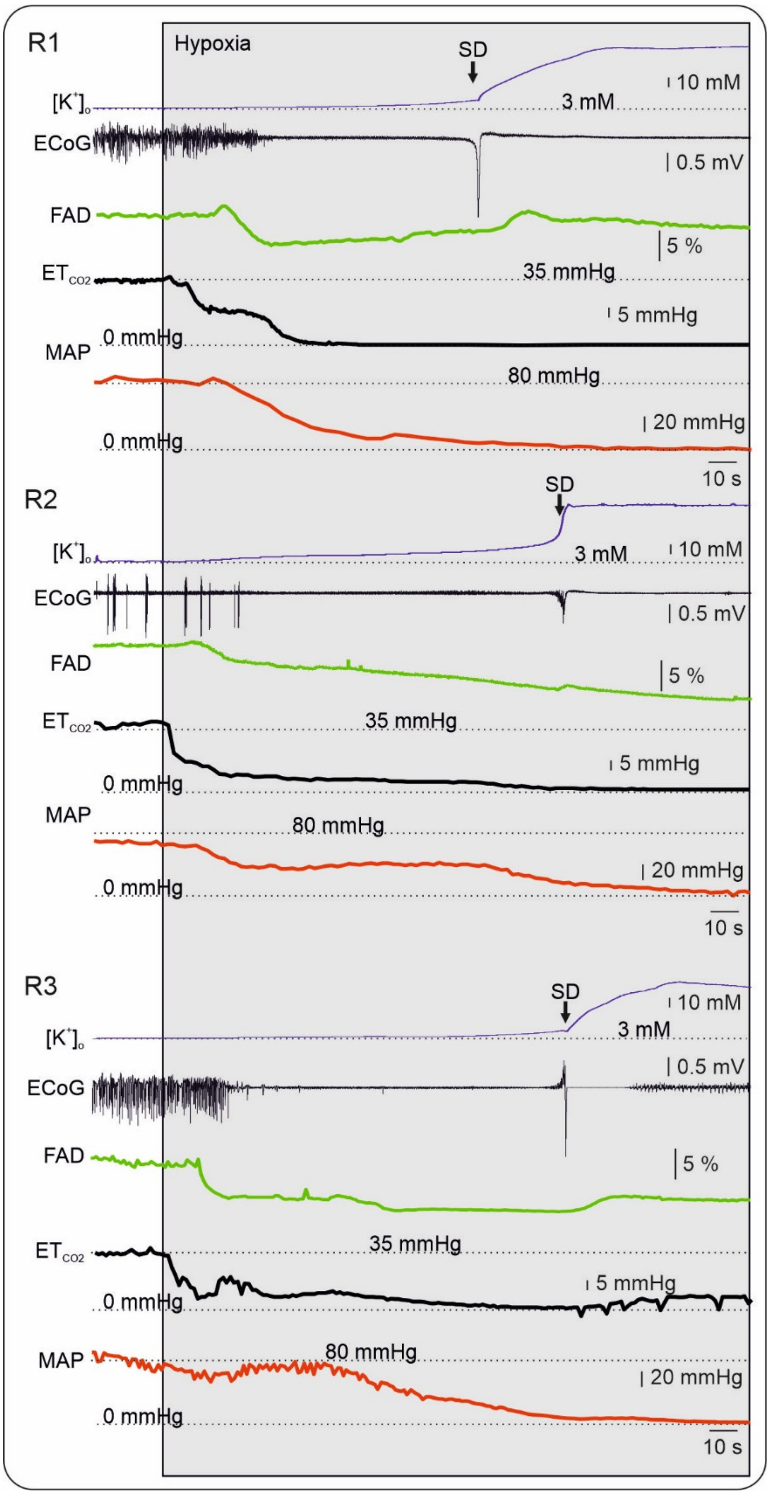

Supplementary Figure 2. Details of the three experiments concerning changes in Flavin adenine dinucleotide (FAD) fluorescence in vivo. R1-R3. Data concerning simultaneous changes in interstitial K+ (blue trace), ECoG-activity (black trace), FAD fluorescence (green trace), end-tidal CO2 (ET_{CO2}, black trace labeled) and middle arterial pressure (MAP, red trace). In all three experiment, a consistent FAD reductive shift was registered during hypoxia. Terminal spreading depolarization (SD, black arrow) occurred after a period of complete circulatory depression as shown by the systemic blood pressure and ETCO2.
